# An Alternative Biventricular Tracking Approach for Assessment of Right Ventricular Strain in Cardiac Amyloidosis

**DOI:** 10.3390/clinpract16090164

**Published:** 2026-09-04

**Authors:** Marina Leitman, Vladimir Tyomkin, Shmuel Fuchs

**Affiliations:** 1 Department of Cardiology, Shamir Medical Center, Zerifin 70300, Israel; 2 Gray Faculty of Medical & Health Sciences, Tel Aviv University, Tel Aviv 69978, Israel

**Keywords:** cardiac amyloidosis, speckle-tracking echocardiography, right ventricular strain, biventricular strain, ventricular mechanics, ventricular deformation

## Abstract

**Background:** Cardiac amyloidosis is characterized by progressive myocardial infiltration leading to ventricular dysfunction. While left ventricular strain assessment is well established, evaluation of right ventricular (RV) mechanics remains challenging because of the ventricle’s complex geometry and limitations of conventional speckle-tracking methods. We aimed to evaluate an alternative approach for RV strain assessment based on a continuous biventricular tracking pathway within the apical four-chamber view. **Methods:** We retrospectively studied 32 patients with cardiac amyloidosis and 32 age-, sex-, rhythm-, and ejection fraction–matched controls. RV free-wall longitudinal strain was measured using a novel investigational biventricular tracking approach from the basal RV free wall through the apex to the basal lateral left ventricular wall and compared with conventional RV strain. Investigational biventricular strain (BVS) was also obtained automatically as a surrogate marker of global ventricular function. Reproducibility was assessed in a subset of 20 patients using intraclass correlation coefficients (ICC), coefficient of variation (CV), and Bland–Altman analysis. **Results:** Investigational RV free-wall strain was significantly reduced in patients with cardiac amyloidosis compared with controls (−15.3 ± 4.1% vs. −21.2 ± 5.4%, *p* < 0.001). Compared with conventional RV strain, the investigational biventricular tracking approach yielded more negative global RV strain values in both patients with amyloidosis (−15.3 ± 4.1% vs. −13.8 ± 4.8%, *p* = 0.003) and controls (−21.2 ± 5.4% vs. −17.4 ± 5.8%, *p* < 0.001). The greatest differences were observed at the apical RV segment. Bland–Altman analysis demonstrated a mean bias of −2.64 percentage points with 95% limits of agreement from −8.41 to +3.12 percentage points. A significant regional deformation pattern along the investigational tracking contour was observed in patients with cardiac amyloidosis (*p* < 0.001), whereas no significant regional variation was observed in controls. BVS correlated strongly with LV global longitudinal strain (r = 0.83 in amyloidosis; r = 0.58 in controls; r = 0.84 overall; all *p* < 0.001). Inter- and intra-observer reproducibility of investigational RV free-wall strain was excellent (ICC 0.976 and 0.985, respectively). **Conclusions:** The investigational biventricular tracking approach is a feasible and highly reproducible method for assessing ventricular longitudinal deformation using a single continuous contour. It provides complementary information while remaining an exploratory investigational technique. Further studies are required to validate its anatomical specificity, clinical applicability, and prognostic value.

## 1. Introduction

Cardiac amyloidosis is characterized by progressive extracellular deposition of amyloid fibrils within the myocardium, leading to increased wall thickness, diastolic dysfunction, and eventually heart failure. Advances in noninvasive imaging and increased clinical awareness have significantly improved the diagnosis of this condition, particularly for transthyretin cardiac amyloidosis (ATTR-CA) [1,2,3,4,5,6,7]. Echocardiography plays a central role in current diagnostic algorithms, complemented by cardiac magnetic resonance imaging and bone scintigraphy [1,2,4,5,6].

Left ventricular (LV) involvement has been extensively studied and represents a cornerstone of echocardiographic evaluation in cardiac amyloidosis. In particular, longitudinal strain analysis has emerged as a key diagnostic tool, with the characteristic pattern of relative apical sparing widely recognized as a hallmark of amyloid infiltration [8,9]. This pattern reflects regional differences in myocardial deformation and has been incorporated into clinical decision-making.

Right ventricular involvement is common in cardiac amyloidosis and has been associated with advanced disease stage and adverse prognosis. Compared with the left ventricle, however, right ventricular involvement has received considerably less attention. Several studies have demonstrated that RV dysfunction is associated with worse outcomes in cardiac amyloidosis [10,11,12,13]. Assessment of RV function remains challenging due to its complex geometry, crescentic shape, and prominent trabeculation. Conventional echocardiographic evaluation relies on parameters such as tricuspid annular plane systolic excursion (TAPSE), tissue Doppler-derived systolic velocity (S′), and, more recently, RV longitudinal strain derived from speckle-tracking echocardiography [14,15]. TAPSE values above 1.7 cm and FAC values above 35% are considered normal [14]. Current recommendations consider absolute RV free-wall longitudinal strain (three-segment) > 20% and RV global longitudinal strain (six-segment) > 17% to be within the normal range [14]. Muraru et al. reported sex-specific lower limits of normal for three-segment RV free-wall longitudinal strain of −22.5% in men and −23.3% in women [16].

Recently, right ventricular-to-pulmonary artery uncoupling was shown to be a powerful predictor of heart failure hospitalization and all-cause mortality in patients with wild-type transthyretin amyloid cardiomyopathy, outperforming several traditional prognostic markers [17].

In a recent meta-analysis, echocardiographic parameters of biventricular deformation, left ventricular systolic and diastolic function, and TAPSE were significantly associated with mortality in patients with cardiac amyloidosis [18].

RV free-wall longitudinal strain, typically obtained from an RV-focused apical four-chamber view, is considered a sensitive marker of RV systolic function. However, this method has important limitations. Unlike the left ventricle, the right ventricle lacks a simple geometric structure, and strain analysis depends on tracking along the RV free wall relative to the interventricular septum. Because of the complex RV geometry and the acute angulation between the RV free wall and the interventricular septum, speckle tracking performance may be more challenging than in the left ventricle, particularly in the apical segments [16,19,20,21].

Importantly, the right and left ventricles are structurally and functionally interconnected. They share myocardial fibers, are enclosed within the same pericardial space, and contract in a coordinated manner in response to common electrical and mechanical activation [22]. These considerations suggest that RV mechanics may also be evaluated within an integrated framework that reflects ventricular interdependence.

Because ventricular interdependence may be particularly relevant in infiltrative cardiomyopathies, integrated assessment of right and left ventricular deformation could provide complementary information regarding ventricular deformation.

We hypothesized that a continuous biventricular tracking pathway could provide an alternative geometric approach for assessing ventricular deformation while preserving continuous tracking across the ventricular apex. We therefore compared this investigational biventricular tracking approach with conventional RV free-wall strain analysis in patients with cardiac amyloidosis and matched controls.

## 2. Materials and Methods

### 2.1. Study Population

We retrospectively enrolled 32 consecutive patients with cardiac amyloidosis (Group 1) who underwent clinically indicated transthoracic echocardiography at our institution between August and December 2025. Twenty-eight patients had transthyretin amyloidosis, and four had light-chain amyloidosis.

The diagnosis of cardiac amyloidosis was established in accordance with current international recommendations. Transthyretin cardiac amyloidosis was diagnosed on the basis of characteristic echocardiographic findings together with positive bone scintigraphy (Perugini grade ≥ 2), provided that monoclonal gammopathy had been excluded. Light-chain (AL) cardiac amyloidosis was confirmed by histopathological demonstration of amyloid deposits with immunohistochemical or immunofluorescent amyloid typing.

All studies were exported for offline analysis. Right ventricular free wall strain was calculated from the apical four-chamber view using both the investigational algorithm and by the conventional method for RV assessment [23].

A control group (Group 2) consisting of 32 subjects matched for age, sex, cardiac rhythm, and left ventricular ejection fraction underwent the same speckle-tracking analysis, including assessment of RV free-wall strain using both the investigational and conventional approaches. Rather than recruiting healthy volunteers, we intentionally selected age- and sex-matched clinical controls, representative of a real-world elderly population referred for routine echocardiography. Consequently, common age-related comorbidities were not considered exclusion criteria, allowing the control cohort to reflect everyday clinical practice.

All consecutive eligible examinations meeting the predefined inclusion criteria were included in the analysis; no studies were excluded because of inadequate image quality or tracking failure.

No formal sample size calculation was performed because the study was exploratory and hypothesis-generating in nature.

Image analyses were performed offline using anonymized echocardiographic datasets without access to clinical records. However, because cardiac amyloidosis has characteristic echocardiographic features, complete blinding to disease status based solely on image appearance was not possible.

### 2.2. Echocardiographic Acquisition and Measurements

All transthoracic echocardiographic examinations were performed using a Vivid E95 ultrasound system (GE Healthcare, Horten, Norway) equipped with a 1.7–4.0 MHz phased-array transducer. Images for speckle-tracking analysis were acquired at frame rates of 50 frames/s or higher.

Standard transthoracic echocardiographic images were acquired in accordance with current recommendations for cardiac chamber quantification [15]. The imaging protocol included parasternal long-axis and short-axis views at the basal, mid-ventricular, and apical levels, together with apical four-, two-, and three-chamber views.

Assessment of left ventricular diastolic function followed contemporary guideline recommendations [24]. The evaluated parameters included transmitral E and A wave velocities, the E/A ratio, E-wave deceleration time, septal and lateral mitral annular E′ velocities obtained by tissue Doppler imaging, and the derived E/E′ ratio.

Left atrial volume was determined using the biplane area–length method:

$$\mathrm{LA}\;\mathrm{volume}=\frac{8}{3\pi }\times \frac{A4ch\times A2ch}{L}$$

where *A*4*ch* and *A*2*ch* denote the left atrial areas measured in the apical four- and two-chamber views, respectively, and *L* represents the shortest left atrial long-axis length. The left atrial volume index (LAVi) was calculated by indexing left atrial volume to body surface area (BSA).

Left ventricular mass (LVM) was estimated using the Devereux equation:

$$LVM=0.8\times 1.04\times [(IVS+PW+LVIDd{)}^{3}-LVIDd^{3}]+0.6\;g$$

where *IVS* is interventricular septal thickness, *PW* is posterior wall thickness, and *LVIDd* is the left ventricular internal diameter at end-diastole. Left ventricular mass index (LVMi) was obtained by indexing LVM to body surface area.

Relative wall thickness (RWT) was calculated as follows:

$$RWT=\frac{2\times PW}{LVIDd}$$

where *LVIDd* is left ventricular internal diameter at end-diastole.

Global longitudinal strain (GLS) was calculated for each patient as previously described [23].

### 2.3. Investigational Right Ventricular Free-Wall Strain Calculation

**Figure 1:**
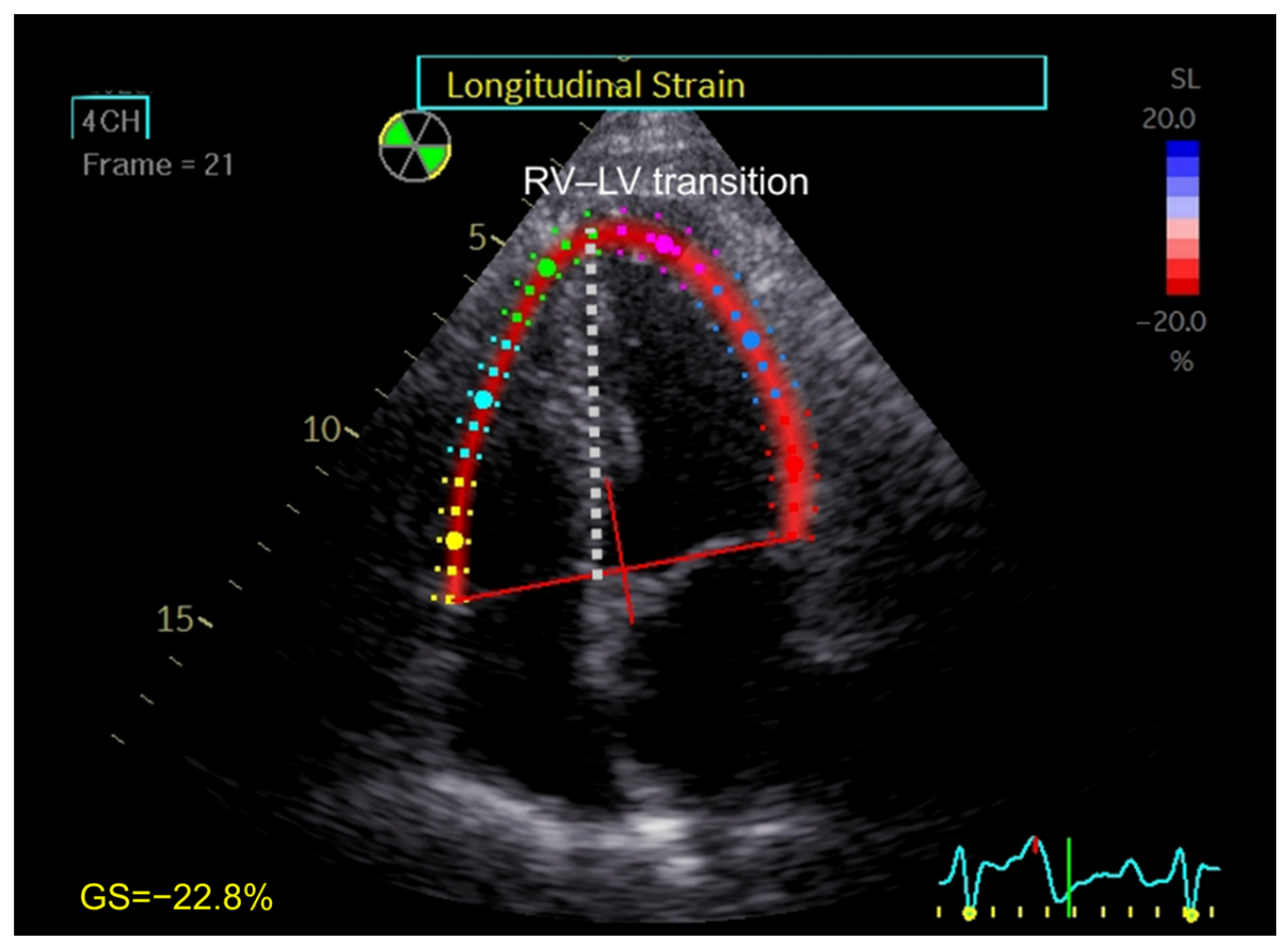
Investigational biventricular tracking approach for right ventricular strain calculation. The region of interest was defined in an optimal apical four-chamber view, extending from the basal right ventricular free wall through the apex to the basal lateral wall of the left ventricle. Right ventricular free-wall strain was calculated as the mean of basal, mid-ventricular, and apical segments along this tracking pathway. In addition, global biventricular longitudinal strain was automatically calculated from the same four-chamber view. The dashed line schematically indicates the anatomical transition between the right and left ventricles. The continuous contour is used for speckle tracking only. Investigational RV free-wall strain is calculated exclusively from the three RV free-wall segments located to the left of the RV–LV transition, whereas the LV lateral-wall segments contribute only to the continuous tracking pathway and to the separately reported BVS.

An optimal apical four-chamber view with clear visualization of both ventricles was selected. A region of interest was defined to include the endocardial contours of both ventricles, enabling a continuous tracking pathway from the basal right ventricular free wall through the apex to the basal lateral wall of the left ventricle (Figure 1).

RV free-wall strain was calculated as the mean of three segments (basal, mid-ventricular, and apical). Segment assignment was performed automatically by the commercial software after contour definition and could not be manually modified. Regional strain values were recorded for each segment. In addition, global biventricular longitudinal strain from the four-chamber view was automatically generated by the software.

Offline speckle-tracking analysis was performed using EchoPAC software (Version 206; GE Healthcare), which permitted manual modification and extension of the tracking region of interest. The investigational contour followed the RV free wall, crossed the apex, and continued along the LV lateral wall. The biventricular strain value was calculated as the arithmetic mean of the predefined segmental strain values and was treated as an investigational composite parameter.

### 2.4. Conventional Right Ventricular Free Wall Strain Calculation

Conventional RV free-wall strain was calculated from the same RV-focused apical four-chamber view as recommended [14,23], using the standard approach based on three RV free-wall segments (basal, mid, and apical), excluding the interventricular septum.

**Figure 2:**
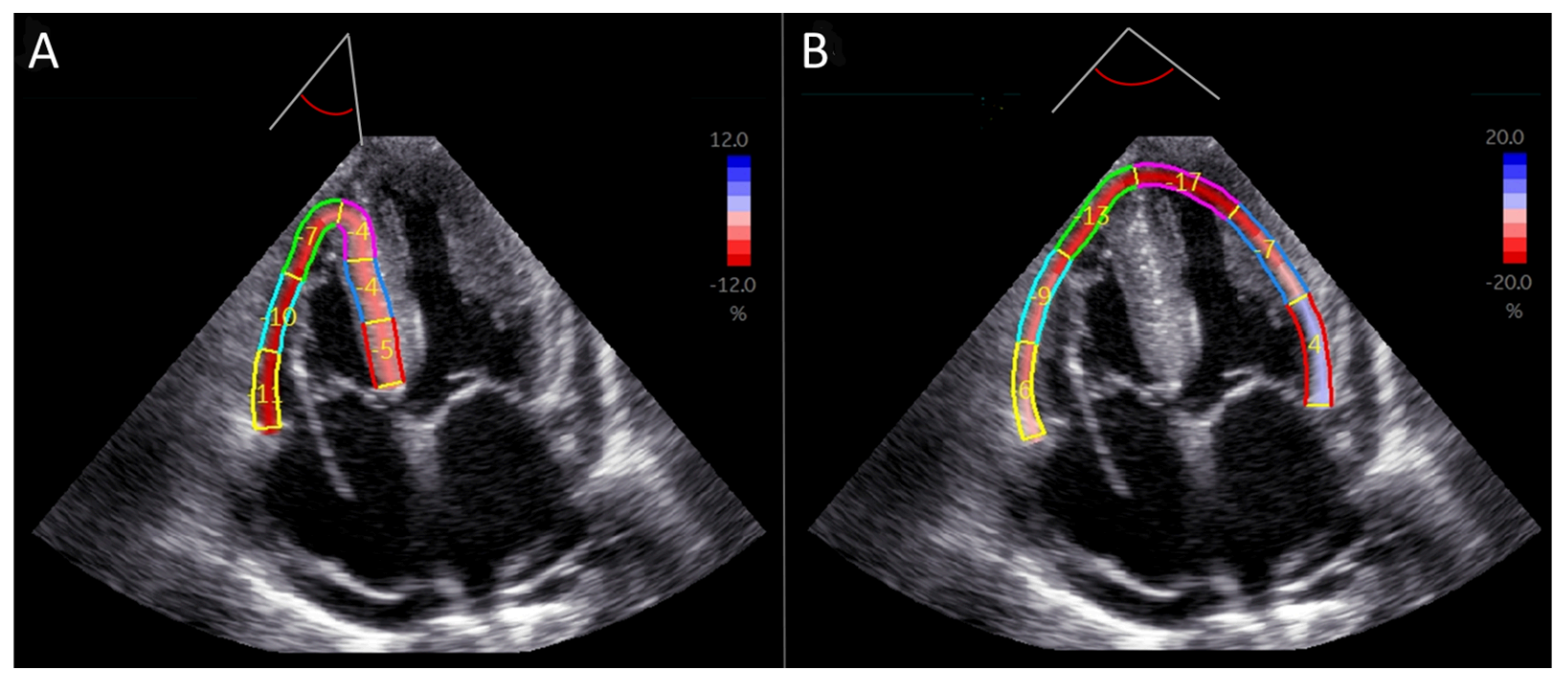
Anatomical and geometric comparison of the conventional and investigational right ventricular strain tracking approaches. (**A**) Conventional method. The region of interest includes both the RV free wall and the interventricular septum. Although RV free-wall strain is reported using only the three RV free-wall segments, speckle tracking requires delineation of both the RV free wall and the septum. (**B**) Investigational method. The tracking contour extends continuously from the basal RV free wall through the apex to the basal LV lateral wall. Within the investigational approach, RV free-wall strain is derived from the three RV free-wall segments (yellow, basal; blue, mid-ventricular; green, apical) as defined by the software after contour placement. The LV lateral-wall segments contribute to the continuous tracking pathway and to the separately reported BVS but are not included in the calculation of the investigational RV free-wall strain average.

The geometric differences between the investigational and conventional right ventricular strain tracking approaches are illustrated in Figure 2.

### 2.5. Reproducibility

A reproducibility analysis was performed in a subgroup of 20 participants, including 10 patients with cardiac amyloidosis and 10 control subjects. Right ventricular strain measurements were repeated to evaluate inter-observer variability between two observers (ML and VT) and intra-observer variability based on repeated analysis by the same observer (ML).

Measurement reproducibility was quantified using the intraclass correlation coefficient [ICC(2,1)] with a two-way random-effects model for absolute agreement. Additional reproducibility indices included the coefficient of variation, Bland–Altman mean bias with 95% limits of agreement (LoA), standard error of measurement (SEM), and repeatability coefficient (RC).

### 2.6. Statistical Analysis

Continuous data are reported as mean ± standard deviation, whereas categorical data are expressed as counts and percentages. Distributional assumptions were evaluated using the Shapiro–Wilk test together with visual assessment of Q–Q plots. Comparisons between the amyloidosis and control groups were performed using independent-samples *t* tests for normally distributed variables and Mann–Whitney *U* tests when normality assumptions were not met. Categorical variables were analyzed using the χ^2^ test or Fisher’s exact test, as appropriate.

Associations between variables were examined using Pearson correlation coefficients for linear relationships and Spearman rank correlation coefficients for ordinal or non-normally distributed data. A two-sided *p* value < 0.05 was considered statistically significant.

Regional differences among basal, mid-ventricular, and apical strain values were assessed using the Friedman test, followed by Bonferroni-adjusted pairwise comparisons between the three ventricular segments (basal vs. mid, basal vs. apical, and mid vs. apical). For descriptive estimation of effect size, the mean paired difference between segments and its corresponding 95% confidence interval were calculated from the paired differences independently of the Friedman analysis.

Except for the Bonferroni-adjusted post hoc pairwise comparisons following the Friedman test, no additional adjustment for multiple comparisons was applied because the study was exploratory.

Statistical analyses were conducted using IBM SPSS Statistics version 28.0 (IBM Corp., Armonk, NY, USA).

### 2.7. Ethics

The study was approved by the Helsinki Ethics Committee of Shamir (Assaf Harofeh) Medical Center (approval No. 0122-25-ASF(V5), 9 February 2026). The echocardiographic examinations included in this retrospective study had been performed between August and December 2025 as part of routine clinical care. Data extraction and research analysis began on 10 February 2026, following receipt of ethics approval. All echocardiographic data were fully de-identified before analysis. Given the retrospective and anonymized design, the requirement for written informed consent was waived.

This observational study is reported in accordance with the STROBE (Strengthening the Reporting of Observational Studies in Epidemiology) Statement. The completed STROBE checklist is provided as Supplementary Materials.

## 3. Results

### 3.1. General Characteristics

Baseline clinical characteristics are presented in Table 1. Most baseline clinical characteristics were similar between the two groups, although chronic pulmonary disease was more common in the control group.

### 3.2. Echocardiographic Characteristics

Table 2 summarizes the echocardiographic findings. Left ventricular ejection fraction did not differ between groups. Compared with controls, patients with amyloidosis had greater left ventricular wall thickness, increased left ventricular mass index, and more marked concentric remodeling.

Indices of diastolic function showed higher E/E′ values in the amyloidosis group, suggesting elevated filling pressures. Tricuspid annular plane systolic excursion was lower in patients with amyloidosis. Pulmonary artery systolic pressure did not differ significantly between groups, although numerically higher values were observed in patients with amyloidosis.

All strain parameters, including conventional RV free-wall strain, investigational RV free-wall strain, and BVS, were significantly reduced in patients with amyloidosis compared with controls (Table 2).

### 3.3. Investigational Global and Regional Right Ventricular Strain Versus Conventional, Table 3

Investigational RV free-wall strain values were more negative than conventional RV free-wall strain values in both study groups, with the largest differences observed in the apical segment.

**Table 3:** Investigational versus conventional right ventricular strain. RVS—Investigational RV free-wall strain.

		Patients with Amyloidosis	
Strain	Investigational method	Conventional method	*p*-value
RVS global, %	−15.3 ± 4.1	−13.8 ± 4.8	0.003
RVS basal, %	−12.6 ± 3.7	−12.1 ± 5.8	0.535
RVS mid-ventricular, %	−15.5 ± 4.5	−15.4 ± 4.8	0.781
RVS apical, %	−17.4 ± 4.9	−13.7 ± 5.2	<0.001
			Control
Strain	Investigational method	Conventional method	*p*-value
RVS global, %	−21.2 ± 5.4	−17.4 ± 5.9	<0.001
RVS basal, %	−20.5 ± 5.5	−17.8 ± 6.4	0.003
RVS mid-ventricular, %	−21.6 ± 6.2	−19.0 ± 6.4	0.001
RVS apical, %	−21.0 ± 5.9	−15.7 ± 6.8	0.001

**Figure 3:**
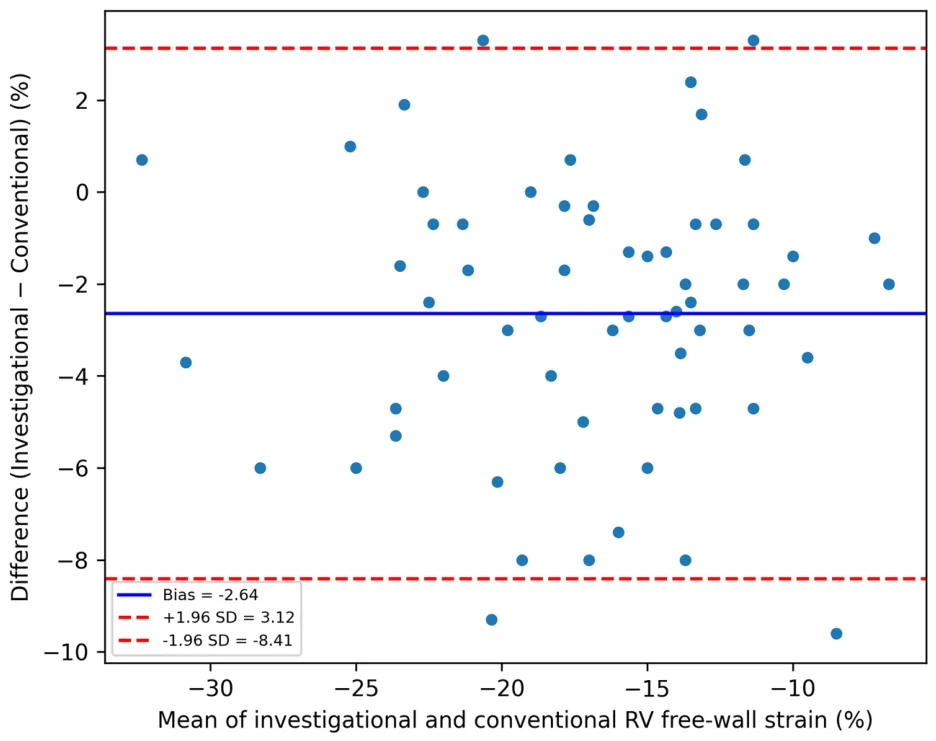
Bland–Altman analysis of investigational and conventional global right ventricular free-wall strain. Each point represents one participant. The x-axis shows the mean of the investigational and conventional RV free-wall strain measurements, and the y-axis shows the difference calculated as investigational minus conventional strain. The solid horizontal line represents the mean bias of −2.64 percentage points, and the dashed horizontal lines represent the 95% limits of agreement, from −8.41 to +3.12 percentage points.

Bland–Altman analysis was performed to evaluate agreement between the two methods at the individual-patient level, Figure 3. The mean bias, calculated as investigational minus conventional strain, was −2.64 percentage points, with 95% limits of agreement from −8.41 to +3.12 percentage points. Although statistically significant differences between the mean values were observed, the relatively wide limits of agreement indicate substantial individual-level differences between measurements.

### 3.4. Regional Differences in Right Ventricular Strain from Base to Apex (Table 4)

Regional differences were assessed using the Friedman test followed by Bonferroni-adjusted pairwise comparisons. In patients with cardiac amyloidosis, significant differences were observed among the basal, mid-ventricular, and apical segments of the investigational contour (Table 4). No significant regional variation was observed in the control group.

**Table 4:** Regional right ventricular free-wall strain measured using the investigational and conventional methods in patients with cardiac amyloidosis and controls.

RV Free-Wall Segment	Investigational Method Amyloidosis (*n* = 32)	Investigational Method Controls (*n* = 32)	Conventional Method Amyloidosis (*n* = 32)	Conventional Method Controls (*n* = 32)
Basal	−12.6 ± 3.7	−20.5 ± 5.5	−12.1 ± 5.8	−17.8 ± 6.4
Mid	−15.5 ± 4.5	−21.6 ± 6.2	−15.4 ± 4.8	−19.0 ± 6.4
Apical	−17.4 ± 4.9	−21.0 ± 5.9	−13.7 ± 5.2	−15.7 ± 6.8
Overall *p*-value *	<0.001	0.269	<0.001	0.005

Values are presented as mean ± SD. * Overall comparison among basal, mid-ventricular, and apical segments performed using the Friedman test for repeated measurements. Post hoc pairwise comparisons were performed using the Bonferroni correction.

**Figure 4:**
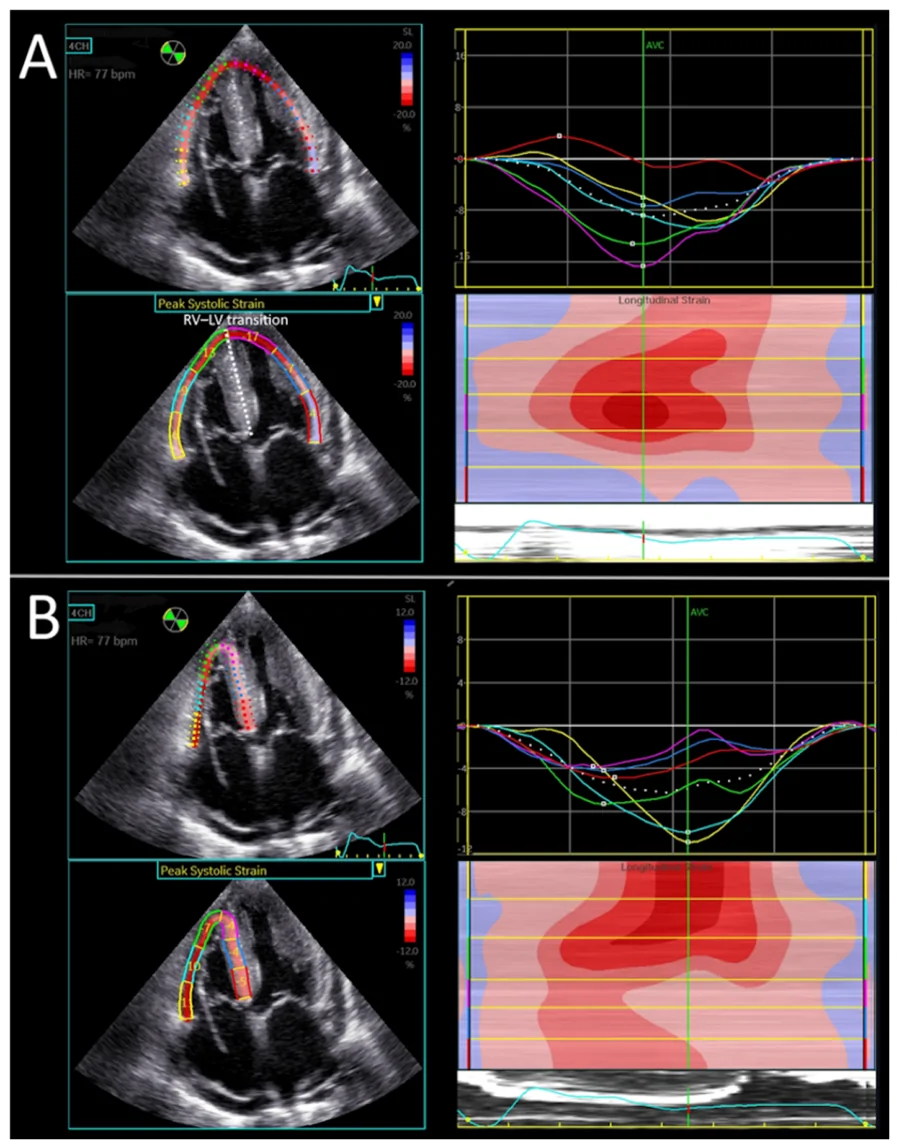
Comparison of investigational and conventional right ventricular strain analysis in a patient with cardiac amyloidosis. Apical four-chamber view demonstrating left ventricular hypertrophy, a small pericardial effusion, and a right ventricular pacemaker lead. (**A**) Investigational biventricular tracking approach. A continuous tracking contour extends from the basal RV free wall through the apex toward the basal LV lateral wall. The dashed line schematically indicates the transition between the RV and LV portions of the investigational contour. Investigational RV free-wall strain is calculated from the three RV free-wall segments defined by the software after contour placement (yellow, basal; blue, mid-ventricular; green, apical), whereas the LV lateral-wall segments contribute only to the continuous tracking pathway and to the separately reported BVS. A regional deformation gradient is observed along the investigational tracking contour. (**B**) Conventional RV free-wall strain analysis. The region of interest includes the RV free wall and the interventricular septum. Although RV free-wall strain is reported using only the three RV free-wall segments, speckle tracking requires delineation of both the RV free wall and the septum. In this example, the regional deformation pattern observed with the investigational tracking approach is not evident using the conventional RV free-wall strain analysis. In this representative patient, both methods yielded a rounded global RV free-wall strain value of −9%, despite differences in contour geometry and segmental strain values. The unrounded values are provided in the figure to avoid a rounding coincidence.

Figure 4 illustrates right ventricular strain calculated by investigational (A) and by conventional (B) methods in patients with cardiac amyloidosis.

### 3.5. Correlations with Conventional RV Strain and GLS

Global investigational RV free-wall strain correlated strongly with conventional RV free-wall strain in patients with cardiac amyloidosis (r = 0.90, *p* < 0.001) and in controls (r = 0.88, *p* < 0.001).

BVS correlated strongly with GLS in patients with cardiac amyloidosis (r = 0.83, *p* < 0.001), moderately in controls (r = 0.58, *p* < 0.001), and strongly in the overall cohort (r = 0.84, *p* < 0.001).

Correlations of investigational and conventional RV free-wall strain with GLS are presented in Table 5. In patients with cardiac amyloidosis, investigational RV free-wall strain showed numerically higher correlation coefficients with GLS than conventional RV strain (r = 0.795 versus r = 0.701). In controls, the correlations were weaker and similar for the two methods (r = 0.360 versus r = 0.378). In the overall cohort, the corresponding correlations were r = 0.694 and r = 0.531. These comparisons are descriptive and do not demonstrate statistical superiority of either correlation.

### 3.6. Reproducibility Analysis

Inter- and intra-observer reproducibility were evaluated in 20 randomly selected examinations. Reliability was assessed using a two-way random-effects, absolute-agreement, single-measure ICC (2,1) with 95% confidence intervals. Agreement and measurement variability were further evaluated using the coefficient of variation, Bland–Altman mean bias and 95% limits of agreement, standard error of measurement, and repeatability coefficient, Table 6.

## 4. Discussion

The present study was designed as a proof-of-concept evaluation of an alternative tracking geometry rather than as validation of a new chamber-specific strain parameter.

### 4.1. Principal Findings

The principal finding of the present study is that a single-contour investigational biventricular tracking approach is technically feasible and demonstrates excellent reproducibility while maintaining a strong association with established RV free-wall strain and LV global longitudinal strain. These findings support the technical feasibility of the proposed approach as an exploratory investigational method rather than establishing incremental clinical utility.

### 4.2. Conceptual Rationale for the Investigational Method

The right ventricle has a complex geometry, and conventional RV free-wall strain is derived from a limited contour that excludes the septum and relies on tracking across regions with abrupt angular transitions. This may reduce tracking continuity, particularly near the ventricular apex.

Unlike conventional RV free-wall strain, which evaluates RV deformation relative to the interventricular septum, the investigational biventricular tracking approach uses a continuous contour extending from the RV free wall through the apex toward the LV lateral wall. The methodological innovation therefore lies in the tracking geometry rather than in modification of the underlying speckle-tracking algorithm (Figure 2).

This geometry provides a continuous tracking pathway across both ventricles and was designed to capture integrated ventricular deformation. Because the investigational contour incorporates both ventricles, the contribution of LV deformation is an inherent characteristic of the proposed method; however, it may also represent a potential source of confounding and should be considered when interpreting the results.

The increasing disagreement between the investigational and conventional methods toward the apex most likely reflects differences in contour geometry rather than differences in the underlying speckle-tracking algorithm. Consistent with the Bland–Altman analysis, the two approaches should therefore be regarded as related, but not interchangeable, measurements.

### 4.3. Relationship Between RV Strain and Biventricular Mechanics

BVS correlated strongly with GLS, indicating a close association between these measurements. The strong correlation between investigational RV free-wall strain and BVS suggests that the investigational biventricular tracking approach is closely related to overall ventricular longitudinal function. At the same time, the Bland–Altman analysis demonstrated that the investigational and conventional methods are related but not interchangeable measurements.

The stronger association between investigational RV free-wall strain and GLS in patients with cardiac amyloidosis is consistent with the integrated nature of the proposed tracking geometry. Because the investigational contour incorporates both right- and left-ventricular myocardium, the contribution of LV deformation is an inherent characteristic of the method but may also represent a potential source of confounding. Therefore, the proposed approach should be regarded as an exploratory composite parameter requiring further validation.

### 4.4. Reproducibility and Technical Considerations

The investigational RV free-wall strain method demonstrated excellent measurement reproducibility but not established anatomical validity. The higher reproducibility observed for BVS likely reflects the inclusion of the LV lateral wall, which generally provides more stable tracking conditions than the thin, trabeculated RV free wall. Accordingly, reproducibility should not be interpreted as evidence of anatomical validity.

The investigational biventricular tracking approach uses a contour geometry that differs from the chamber-specific geometries for which commercial speckle-tracking software was originally developed. Although longitudinal strain is calculated locally from changes in segment length and is therefore independent of the opposite anatomical orientation of the RV and LV walls, the effects of contour curvature, segment smoothing, spatial regularization, and drift correction in this non-standard geometry have not been independently validated. Consequently, BVS should be regarded as an exploratory composite parameter rather than an established chamber-specific strain measurement.

### 4.5. Right Ventricular Involvement in Cardiac Amyloidosis

Although left ventricular involvement is the hallmark of cardiac amyloidosis, right ventricular dysfunction is common and carries important prognostic implications. Previous studies have shown that impaired right ventricular function is associated with more advanced disease and worse clinical outcomes [10,11,12,13]. However, compared with the left ventricle, assessment of right ventricular mechanics remains more challenging because of the complex geometry of the right ventricle and technical limitations of conventional imaging approaches.

The investigational biventricular tracking approach provides a single-contour visualization of ventricular deformation within one apical four-chamber view. As illustrated in Figure 4, it facilitates simultaneous visualization of right- and left-ventricular deformation while preserving separate reporting of investigational RV free-wall strain and the exploratory biventricular strain parameter. This integrated display may facilitate visual assessment of ventricular involvement; however, its clinical utility remains to be established. These findings are consistent with previous observations that cardiac amyloidosis affects both ventricles and support further investigation of integrated biventricular assessment in this disease [25,26].

### 4.6. Clinical Implications

The investigational biventricular tracking approach represents a technically feasible investigational method for assessing ventricular deformation using a single continuous contour. Although it should not be considered a replacement for conventional RV free-wall strain analysis, it may provide complementary information regarding ventricular deformation within an integrated mechanical framework [27,28].

Because the investigational contour incorporates both right- and left-ventricular myocardium, the contribution of left ventricular deformation is an inherent characteristic of the proposed method and may also represent a source of confounding, particularly in diseases with marked left ventricular dysfunction. Accordingly, the exploratory biventricular strain parameter should be interpreted as a composite measure requiring further validation.

Conventional RV free-wall strain analysis remains technically challenging because of the complex geometry of the right ventricle and its dependence on image quality and tracking performance [29,30]. Although the present study focused on cardiac amyloidosis, the proposed tracking geometry is not disease-specific and could be investigated in other conditions characterized by ventricular interdependence or biventricular remodeling. Whether this approach provides incremental diagnostic, clinical, or prognostic value remains to be determined in future prospective studies.

### 4.7. Limitations

This study has several limitations. It was a single-center study with a relatively small sample size, which may limit the generalizability of the findings. The investigational biventricular tracking approach was evaluated using a single vendor platform and has not been validated against independent reference standards such as cardiac magnetic resonance imaging, right ventricular ejection fraction, fractional area change, or clinical outcomes. Because the proposed tracking contour represents a non-standard geometry, potential averaging of adjacent RV and LV myocardium at the ventricular apex cannot be excluded and requires further validation using independent imaging modalities or dedicated tracking software. Although the method demonstrated excellent reproducibility, it remains partially operator-dependent because of manual contour definition. Therefore, the present findings should be regarded as exploratory and hypothesis-generating.

Only four patients with AL cardiac amyloidosis were included; therefore, no subtype-specific analyses were performed, and the findings apply only to the pooled cardiac amyloidosis cohort. In addition, the study population consisted of symptomatic patients referred for clinical evaluation, and the results should not be extrapolated to patients with early or asymptomatic disease.

The control cohort consisted of clinical rather than healthy subjects and had a higher prevalence of chronic pulmonary disease, which may have influenced RV deformation measurements. Although pulmonary artery systolic pressure did not differ significantly between groups, residual confounding by RV afterload cannot be excluded.

## 5. Conclusions

The investigational biventricular tracking approach represents a feasible, reproducible investigational approach for assessing ventricular longitudinal deformation using a single continuous contour. The proposed approach provides complementary information to conventional strain analysis and demonstrates the technical feasibility of single-contour biventricular strain assessment using commercially available speckle-tracking software. Further prospective studies are required to determine whether this approach has clinical or prognostic utility.

## Figures and Tables

**Table 1:** Demographic and clinical characteristics of patients.

Variable	Amyloidosis Patients	Control Patients	*p*-Value
Total number of patients	32	32	1.000
Age, years	83.0 ± 6.0	82.6 ± 6.8	0.817
Male sex, *n* (%)	25 (78.1)	25 (78.1)	1.000
Weight, kg	70.4 ± 10.9	76.7 ± 14.4	0.057
Height, cm	167.2 ± 8.6	169.2 ± 8.2	0.337
BMI, kg/m^2^	25.2 ± 3.2	26.7 ± 4.3	0.117
BSA, m^2^	1.80 ± 0.17	1.87 ± 0.20	0.107
Atrial fibrillation during echo examination, *n* (%)	11 (34.4)	11 (34.4)	1.000
Hypertension, *n* (%)	22 (68.8)	26 (81.3)	0.387
Chronic kidney disease, *n* (%)	18 (56.3)	13 (40.6)	0.317
Diabetes, *n* (%)	14 (43.8)	10 (31.3)	0.439
Ischemic heart disease, *n* (%)	8 (25.0)	6 (18.8)	0.763
History of atrial fibrillation, *n* (%)	20 (62.5)	14 (43.8)	0.210
Chronic pulmonary disease, *n* (%)	1 (3.1)	11 (34.4)	0.003
History of malignancy, *n* (%)	7 (21.9)	9 (28.1)	0.774
NYHA functional class	3 (2–3)	3 (2–3)	0.372

Values are presented as mean ± SD, median (interquartile range), or *n* (%), as appropriate. Continuous variables were compared using the independent-samples *t*-test or Welch’s *t*-test, as appropriate; NYHA functional class was compared using the Mann–Whitney U test; and categorical variables were compared using the χ^2^ test or Fisher’s exact test, as appropriate.

**Table 2:** Echocardiography parameters of study patients.

Parameter	Amyloidosis Patients	Control Patients	*p*-Value
Left atrial volume index, mL/m^2^	54.9 ± 15.1	46.9 ± 20.6	0.09
Left ventricle end-diastolic diameter, cm	4.3 ± 0.5	4.6 ± 0.6	0.08
Left ventricle end-systolic diameter, cm	2.8 ± 0.5	2.7 ± 0.5	0.45
Interventricular septum thickness, cm	1.7 ± 0.3	1.4 ± 0.2	0.001
Posterior wall thickness, cm	1.4 ± 0.2	1.1 ± 0.2	<0.001
Left ventricular mass index, g/m^2^	148 ± 27.4	112 ± 36.3	0.001
Relative wall thickness	0.64 ± 0.13	0.48 ± 0.1	<0.001
Ejection fraction, %	53.9 ± 4.7	54.1 ± 2.8	0.82
E deceleration time	184.8 ± 52.5	197.6 ± 62.7	0.38
E/e’	19.1 ± 7.9	13.6 ± 6.9	0.004
TAPSE, cm	1.6 ± 0.4	2.1 ± 0.4	<0.001
Pulmonary artery pressure, mmHg	45.5 ± 14.9	39.9 ± 11.2	0.1
Global longitudinal strain, %	−10.4 ± 3.2	−20.3 ± 2.6	<0.001
Biventricular strain, %	−11.7 ± 3.7	−18.3 ± 3.8	<0.001
Conventional right ventricular strain, %	−13.8 ± 4.8	−17.4 ± 5.9	0.01
Investigational right ventricular strain, %	−15.3 ± 4.1	−21.2 ± 5.4	<0.001
Heart rate during the echo exam, beats/min	72.9 ± 12.7	74.7 ± 13.2	0.58
Frame rate used for speckle-tracking analysis, frames/s	55.9 ± 2.9	56.5 ± 3.2	0.47

**Table 5:** Correlations of investigational and conventional RV free-wall strain with LV global longitudinal strain.

Group	Investigational RV Strain	*p*-Value	Conventional RV Strain	*p*-Value
Amyloidosis, *n* = 32	r = 0.795	<0.001	r = 0.701	<0.001
Controls, *n* = 32	r = 0.360	0.043	r = 0.378	0.033
Overall, *n* = 64	r = 0.694	<0.001	r = 0.531	<0.001

**Table 6:** Reproducibility of investigational RV free-wall strain and BVS.

Parameter	Type	ICC(2,1) (95% CI)	CV (%)	Mean Bias (Percentage Points)	95% Limits of Agreement (Percentage Points)	SEM	RC
Investigational RV strain	Inter-observer	0.976 (0.939–0.991)	4.2	+0.44	−1.73 to +2.60	0.78	2.17
	Intra-observer	0.985 (0.962–0.994)	3.6	+0.24	−1.61 to +2.09	0.67	1.85
Biventricular strain	Inter-observer	0.994 (0.984–0.998)	2.7	+0.09	−1.08 to +1.25	0.42	1.16
	Intra-observer	0.991 (0.978–0.996)	3.4	−0.11	−1.54 to +1.33	0.52	1.43

CI, confidence interval; SEM, standard error of measurement; RC, repeatability coefficient; Lo A, limits of agreement. ICC values were calculated using a two-way random-effects, absolute-agreement, single-measure model [ICC (2,1)]. Bias was calculated as the second measurement minus the first measurement. All reproducibility indices were calculated from the same set of 20 paired measurements.

## Data Availability

The raw data supporting the conclusions of this article will be made available by the authors on request.

## Supplementary Materials

The following supporting information can be downloaded at https://www.mdpi.com/article/10.3390/clinpract16090164/s1: Figure S1: Participant flow diagram. Flow diagram illustrating the selection of the cardiac amyloidosis and age- and sex-matched clinical control cohorts included in the study, together with the randomly selected subset used for the reproducibility analysis. No eligible examinations were excluded because of inadequate image quality or tracking failure; Table S1: STROBE Checklist.
