# Supplementary material for: An Alternative Biventricular Tracking Approach for Assessment of Right Ventricular Strain in Cardiac Amyloidosis
Source: Clin Pract. 2026 Sep 4;16(9):164. doi: 10.3390/clinpract16090164 (PMC13605196; doi:10.3390/clinpract16090164)
Supplement: Supplementary file 1 [file clinpract-16-00164-s001.zip › clinpract-4426646-supplementary.pdf]

Supplementary files

Supplementary Figure S1 – Participant Flow Diagram

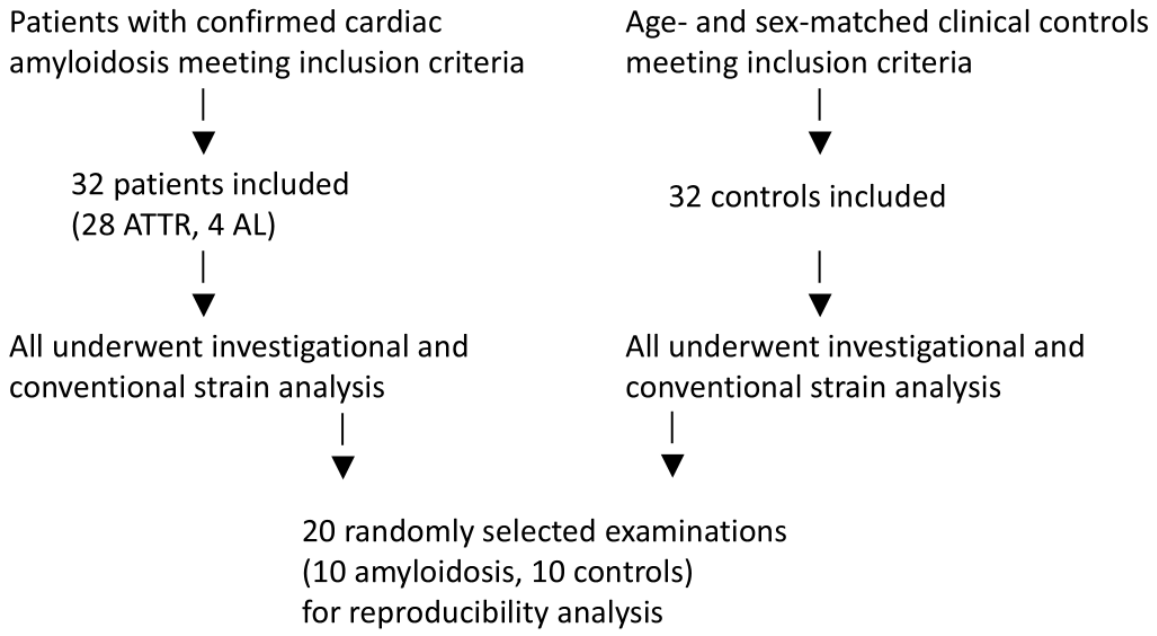

Supplementary Figure S1. Participant flow diagram. *Flow diagram illustrating the selection of the cardiac amyloidosis and age- and sex-matched clinical control cohorts included in the study, together with the randomly selected subset used for the reproducibility analysis. No eligible examinations were excluded because of inadequate image quality or tracking failure.*

Supplementary Table S1 – STROBE Checklist

STROBE item	Recommendation	Page(s)
**Title and Abstract**		
1a	Indicate the study design in the title or abstract	1–2
1b	Provide an informative and balanced abstract	2
**Introduction**		
2	Explain scientific background and rationale	3–4
3	State specific objectives and hypotheses	4
**Methods**		
4	Present key elements of study design	4
5	Describe setting, location and dates	4
6a	Eligibility criteria and participant selection	4–5
6b	Matching criteria for controls	4
7	Define outcomes, exposures, predictors and variables	5–8
8	Describe data sources and measurement methods	5–8
9	Describe efforts to address bias	5
10	Explain study size	5
11	Explain handling of quantitative variables	8
12a	Describe all statistical methods	8
12b	Methods for subgroup analyses	Not applicable
12c	Explain handling of missing data	4–5
12d	Matching of cases and controls	4
12e	Sensitivity analyses	Not performed
**Results**		

STROBE item	Recommendation	Page(s)
13a	Numbers of participants at each stage	9
13b	Reasons for non-participation	4–5 (none)
13c	Flow diagram	Supplementary Figure S1
14a	Participant characteristics	9–10 (Table 1)
14b	Missing data	None
15	Outcome data	10–14
16	Main results	10–14
17	Other analyses	13–14
**Discussion**		
18	Summarize key results	14
19	Discuss limitations	17
20	Provide cautious overall interpretation	14–17
21	Discuss generalizability	17
**Other information**		
22	Funding	Funding section (or "No external funding")
23	Ethics approval	8
